# Mast Cell Deficiency Protects Mice from Surgery-Induced Neuroinflammation

**DOI:** 10.1155/2020/1921826

**Published:** 2020-08-01

**Authors:** Xiang Zhang, Hongquan Dong, Fei Wang, Jun Zhang

**Affiliations:** ^1^Department of Anesthesiology, Fudan University Shanghai Cancer Center, Department of Oncology, Shanghai Medical College, Fudan University, Shanghai 200032, China; ^2^Department of Anesthesiology, The First Affiliated Hospital of Nanjing Medical University, Nanjing, Jiangsu 210029, China

## Abstract

Neuroinflammation plays a key role in the occurrence and development of neurodegenerative diseases. Microglia, the resident immune cells in the brain, have been recognized to contribute to neuroinflammation. Previous studies have shown that activated mast cells may be involved in surgery-induced neuroinflammation and neuronal apoptosis by using pharmacological methods. This study is aimed at ascertaining the exactly role of mast cells on neuroinflammation with the mast cell-deficient mice. Adult male C57BL6/J wild-type (WT) and mast cell-deficient (C57BL6/J KitWsh/Wsh (Wsh)) mice underwent tibial fracture surgery. Blood-brain barrier (BBB) breakdown, microglial activation, and neuroinflammatory levels were examined at 1 day after surgery. Surgery-induced BBB breakdown, microglial activation, and neuroinflammatory levels were significantly, pharmacologically reduced using a mast cell stabilizer, cromolyn sodium in WT mice (*P* < 0.05). These results were reproduced with mast cell deficiency. WT mice administered intraventricularly with cromolyn exhibited reduced BBB breakdown, microglial activation, and neuroinflammatory levels versus vehicle (*P* < 0.05). But there was no effect of cromolyn versus vehicle in Wsh mice, clarifying the specificity of cromolyn on brain mast cells. These findings demonstrated that activated mast cells promote surgery-induced BBB breakdown and neuroinflammation in mice, and open up a new therapeutic target for neuroinflammation-related diseases.

## 1. Introduction

It is widely recognized that neuroinflammation plays an important role in CNS disorders, such as neurodegenerative diseases [1]. The induction and acceleration of neuroinflammation seem to depend on the communication between microglia, neurons, and immune cells. However, little is known about the microglial immune cell connection thus far. Animal models of peripheral surgical intervention, such as tibial fracture, trigger neuroinflammation in the brain, which is frequently used as an animal model for studying neurodegeneration [2].

Microglia are primary resident immune cells in the brain. Accumulating reports have defined microglial activation as an important element of neuroinflammation. Microglia could be classified into two states: a M1 reactive phenotype initiating an inflammatory response and M2 phenotype with an anti-inflammatory role. Overactivation of microglia produces numerous inflammatory mediators, leading to neuronal damage and brain injury. Hence, restraining microglia-induced excessive inflammatory response may improve neurodegenerative diseases.

Emerging evidence indicates that microglia respond to inflammatory mediators released by other immune cells like mast cells. Mast cells are located in the brain side of blood-brain barrier (BBB). Under various stimulations, mast cells secrete numerous mediators, including proteases, vasoactive amines, tryptase, and histamine. Our previous studies have demonstrated that these inflammatory mediators could evoke microglial activation. Mast cell stabilizer cromolyn limited microglial activation by inhibiting mast cell degranulation [3]. Notably, meningeal mast cells are able to recruit different types of immune cells to the brain by penetrating BBB and breaking its integrity. The exact effect of mast cells on microglia has not been fully illuminated to date. Furthermore, there is little evidence about the involvement of mast cells in tibial fracture-induced neuroinflammation. The aim of this study is to use genetically mast cell-deficient mice to clarify the role of mast cells on the microglial activation and neuroinflammation.

## 2. Materials and Methods

### 2.1. Animals

All experimental procedures were approved by the Institutional Animal Care and Use Committee of Fudan University and conducted in accordance with the policies of institutional guidelines on the care and use of laboratory animals. Male mice were housed under specific pathogen-free conditions (40% humidity; 22.0 ± 1.0°C temperature), five animals per cage during breeding and the experiments, with free access to normal food and water.

C57BL6/J KitWsh/Wsh (Wsh) mice, the mast cell-deficient mice used in our study, were obtained from Model Animal Research Center of Nanjing University. Adult Wsh mice are profoundly mast cell-deficient. The Wsh is a mutant allele at the W (c-kit) locus of mice. Mice of Wsh/Wsh genotype have white hairs and black eyes, and show a remarkable depletion of mast cells.

### 2.2. Model of Surgery

Tibial fracture surgery model was received as previously described [4]. An incision under the right knee was made after sevoflurane anesthesia and implanted 26 G needle into medullary canal of the tibia. Tibial fracture was then generated in the midshaft.

### 2.3. Stereotaxic Injection of Cromolyn Sodium

In one set of experiments, two groups of mice were assigned to inject either sterile saline (vehicle) or cromolyn sodium (Sigma) (75 *μ*g in 2 *μ*l saline) into the ventricle randomly. The surface of the dura is defined as zero reference, -0.6 mm anterior/posterior, +1 mm medial/lateral, and 2.4 mm ventral. After the same anesthesia 30 minutes before surgery, stereotaxic injection was performed with 32-gauge needle [5]. Over a 2-minute period, 2 *μ*l of cromolyn sodium or vehicle was injected, and the needle was left in place for 2 minutes further. The needle was then slowly retracted and the wound was sutured.

### 2.4. Immunohistochemical and Immunofluorescence Analysis

The brain sections of the mice were obtained as previously described [5, 6]. Then, the brain sections were tested through immunohistochemistry and immunofluorescence. The primary antibodies used in this experiment were rabbit anti-tryptase (1 : 100), rabbit anti-Iba1 (1 : 200), and anti-rabbit secondary antibodies (1 : 200).

## 3. PCR

Real-time PCR is used to assess the expression of CD86 and CD32, which are the two kinds of M1 phenotype markers in the hippocampus as previously described [6].

### 3.1. TNF-*α* and IL-1*β* Assay

The frozen hippocampus tissues were rinsed with PBS to remove excess blood. Tissues were then chopped into 1-2 mm pieces and homogenized in 100 mg tissue/ml cold PBS. The homogenized materials were centrifuged at 12,000 *g* for 15 min, and the cleared supernatant was collected for analysis. Total protein levels were determined using a BCA protein assay reagent kit (Beyotime). The expression of tumor necrosis factor-*α* (TNF-*α*) and interleukin-1*β* (IL-1*β*) in the hippocampus was measured using ELISA kits according to the manufacturer's instructions (R&D Systems). Briefly, 50 *μ*l standard or sample was added to each well and incubated at room temperature for 2 hours. After five washes, 100 *μ*l mouse TNF-*α* or IL-1*β* conjugate was added to each well and incubated at room temperature for an additional 2 hours. After five washes, 100 *μ*l substrate solution was added for 30 min, followed by 100 *μ*l stop solution. Finally, a microplate reader was used to measure the optical density (OD) of each well at 450 nm.

### 3.2. Statistical Analysis

All dates are presented as the means ± SEM. Comparisons between two groups were determined by Students' unpaired *t*-test. Comparisons among multiple groups were performed using one-way ANOVA. *P* < 0.05 was defined as significantly different.

## 4. Results

### 4.1. Surgery-Induced Mast Cell Degranulation and Neuroinflammation in the Hippocampus in WT Mice

**Figure 1:**
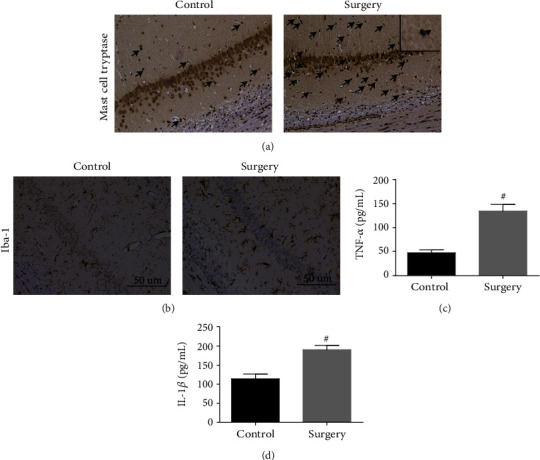
Surgery-induced mast cell degranulation and neuroinflammation in the hippocampus. (a) Mast cell tryptase in area CA1 of the hippocampus was detected by immunostaining. Scale bar is 50 *μ*m. (b) Immunostaining was used to test Iba1 in area CA1 of the hippocampus. Scale bar is 50 *μ*m. (c, d) ELISA was used to detect the expression of proinflammatory factors TNF-*α* and IL-1*β*. ^#^*P* < 0.05 versus control group. Data are presented as the mean ± SEM (*n* = 5).

To examine the relationship between mast cells and surgery-induced neuroinflammation, we first quantified brain mast cells in the hippocampus at 1 day after surgery. And we found a significant increasing number of mast cells in the hippocampus CA1 area after surgery (Figure 1(a)). Meanwhile, a notable microglial activation in the hippocampus was also induced by surgery (Figure 1(b)). To observe the level of neuroinflammation, we also tested the expression of proinflammatory cytokines in the hippocampus by ELISA. And as the same results with our previous studies, surgery induced the obvious increase of TNF-*α* and IL-1*β* (Figure 1(c)). These results indicate that mast cells may be involved in surgery-induced neuroinflammation.

### 4.2. Baseline Levels of Neuroinflammatory Markers Were Unchanged by Mast Cell Deficiency

**Figure 2:**
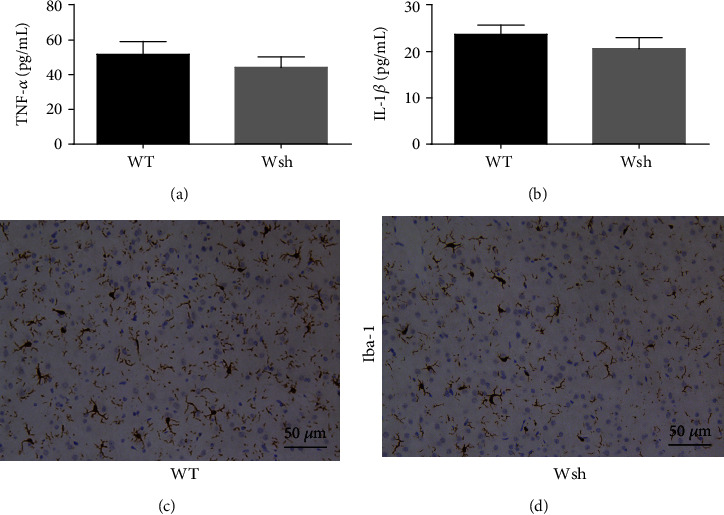
Baseline levels of neuroinflammatory markers were unaffected by mast cell deficiency. (a, b) ELISA was used to detect the expression of proinflammatory factors TNF-*α* and IL-1*β*. (c, d) Iba1 in area CA1 of the hippocampus was detected by immunostaining. Scale bar is 50 *μ*m. Data are presented as the mean ± SEM (*n* = 5).

With the potential proinflammatory role of mediators derived from mast cells between the two strains of mice used, we detected the baseline levels of inflammatory markers in WT and Wsh mice. As shown in Figure 2, there were no statistical differences of proinflammatory factors (TNF-*α* and IL-1*β*) and microglial activation between WT and Wsh mice. These data suggest that mast cell deficiency did not increase the severity of inherent inflammatory levels in the hippocampus.

### 4.3. Mast Cells Contribute to Surgery-Induced BBB Breakdown

**Figure 3:**
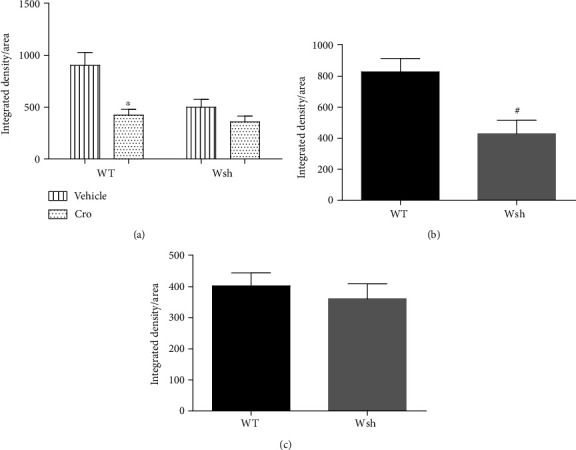
IgG leakage into the brain was inhibited by mast cell stabilization and deficiency. (a) The level of IgG in the hippocampus was detected by immunostaining in mice pretreated with cromolyn sodium versus vehicle. (b, c) In a separate experiments, IgG was measured at 1 day and 3 days after surgery. ^∗^*P* < 0.05 versus WT vehicle. ^#^*P* < 0.05 versus WT group. Data are presented as the mean ± SEM (*n* = 5).

Our previous studies have showed that mast cells could increase BBB permeability after peripheral surgery in the rat model [7, 8]. To test the integrity of BBB, we measured endogenous IgG in the hippocampus. Cromolyn sodium significantly reduced IgG levels compared with vehicle treatment in the hippocampus of WT mice at 1 day after surgery (*P* < 0.05). To confirm the specific effects of cromolyn sodium on mast cells, we repeated the experiments in Wsh mice. Compared with vehicle treatment, the reduction of IgG has no significant difference in cromolyn-treated Wsh mice (Figure 3(a)). The separate study comparing WT with Wsh mice showed that the level of IgG in the hippocampus was reduced in Wsh mice significantly compared with WT mice at 1 day after surgery (*P* < 0.05, Figure 3(b)), but not at 3 days after surgery (Figure 3(c)). The above dates suggest that mast cells have a detrimental role on BBB after surgery, inducing the broken of integrity.

### 4.4. Microglial Activation Was Inhibited by Mast Cell Stabilization and Deficiency

**Figure 4:**
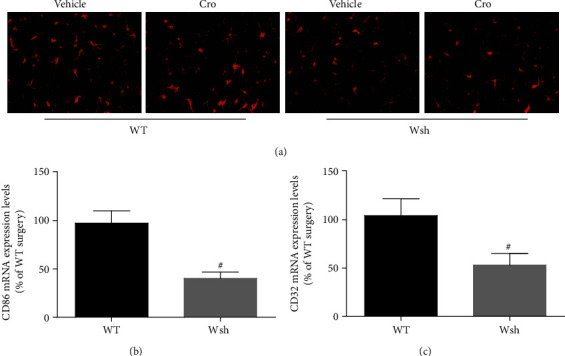
Microglial activation in the hippocampus was inhibited by mast cell stabilization and deficiency. (a) Iba1 in area CA1 of the hippocampus was detected by immunostaining. Scale bar is 25 *μ*m. (b, c) Expression levels of CD86 and CD32 (M1 phenotype markers) at 1 day after surgery were measured by real-time PCR. ^#^*P* < 0.05 versus WT group. Data are presented as the mean ± SEM (*n* = 5).

Our previous study has indicated the potential involvement of mast cells in microglial activation after surgery [6]. Consistent with this, microglial activation was significantly inhibited after cromolyn sodium treatment in WT mice (*P* < 0.05). But the reduction was not significant in Wsh mice (Figure 4(a)). In a separate study, to further clarify the effect of mast cells on microglia, we detected the expression of M1 phenotype markers (CD86 and CD32) in the hippocampus at 1 day after surgery. The levels of CD86 and CD32 in the hippocampus were significantly reduced in Wsh mice compared with WT mice at 1 day after surgery (*P* < 0.05) (Figures 4(b) and 4(c)). These results suggest that surgery-induced microglial activation can be inhibited by mast cell stabilization and deficiency.

### 4.5. Proinflammatory Cytokine Secretion Was Inhibited by Mast Cell Stabilization and Deficiency

**Figure 5:**
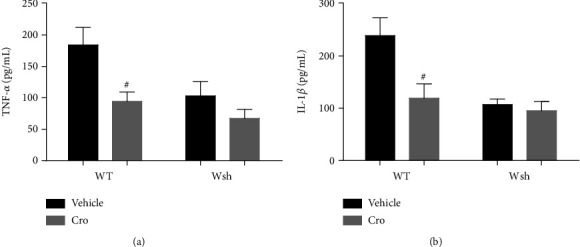
Proinflammatory cytokine secretion in the hippocampus was altered by mast cell stabilization and deficiency. The levels of TNF-*α* (a) and IL-1*β* (b) were detected by ELISA. ^#^*P* < 0.05 compared with WT vehicle. Data are presented as the mean ± SEM (*n* = 5).

Mast cells are recognized to store preformed TNF-*α* and IL-1*β*, and may be the early resource of these cytokines [9, 10]. But there was no evident difference between the baseline levels of WT and Wsh mice (Figure 2). Since neuroinflammation mediated by microglia mainly depends on proinflammatory factors released from activated microglia, we detected the levels of proinflammatory factors TNF-*α* and IL-1*β* at 1 day after surgery by ELISA. As shown in Figure 5, compared with vehicle treatment in WT mice, cromolyn sodium treatment decreased the expression of TNF-*α* and IL-1*β* significantly (*P* < 0.05). And the reduction has no significant difference between cromolyn-treated Wsh mice versus vehicle treatment. These results suggest that mast cells participate in surgery-induced neuroinflammatory cytokine secretion.

## 5. Discussion

As the increasing life span and expectancy, neurodegenerative diseases have turned into a health and economic problem. Neuroinflammation has recognized as the pathogenic mechanism of various neurodegenerative diseases, such as Alzheimer's disease (AD) and Parkinson's disease (PD) [11, 12]. Our previous studies have raised the potential regulatory role of mast cells on neuroinflammation [3]. However, the exact effect of mast cells on surgery-induced neuroinflammation is still unclear. In this study, we clarified the involvement of mast cells on postoperative neuroinflammation with mast cell-deficient mice (Wsh). The major findings are that mast cells are involved in BBB breakdown, microglial activation, and neuroinflammation.

Neurodegenerative diseases such as AD, amyotrophic lateral sclerosis, PD, and postoperative cognitive dysfunction are among the most pressing problems of the developed societies with aging populations. And these diseases are always related with adverse clinical outcomes including heavier burden of economy, low quality of life, and high mortality rate. Furthermore, accumulating evidence has shown that neuroinflammation contributes to the development of neurodegenerative diseases [11, 13]. In this study, we use the mouse model of tibial fracture surgery to explore the exact involvement of mast cells in neuroinflammation. Systemic inflammation evoked by surgery can cause organ dysfunction or failure. Neuroinflammation after surgery has been recognized as the mechanism resulting in neuronal apoptosis, synaptic impairment, and impaired neurogenesis. Studies have shown that uninhibited inflammatory response induced by higher stress and surgical trauma correlates with longer hospital stay [14]. In the present study, we found that, as the same results with previous tests, peripheral surgery could induce the microglial activation and proinflammatory cytokine secretion at 1 day after surgery. And the level of neuroinflammation was linked with mast cell degranulation. Hence, activated mast cells may be involved in surgery-induced neuroinflammation. Kempuraj et al. highlighted the possible role of mast cell activation in neuroinflammation, BBB disruption, and AD pathogenesis. Stress conditions activate mast cells to release prestored and newly synthesized inflammatory mediators and induce increased BBB permeability, recruitment of immune and inflammatory cells into the brain, and neuroinflammation [15]. Similar to our previous studies, Skaper et al. also reviewed mast cell-glia crosstalk and its involvement in neuroinflammation; they indicated the dialogue contributes to accelerate disease progression and become exaggerated with aging and increased cell sensitivity to stress [16]. Nautiyal et al. pointed out that the brain mast cells may be involved in anxiety-like behavior [17]. An animal study recognizes mast cells as the effect cells of neuroinflammation and BBB breakdown in mice after transient middle cerebral artery occlusion [5]. About 1/3 of mastocytosis patients have neuropsychological symptoms [18]. Inhibition of mast cell activation by masitinib can improve the syndrome in ADs [19]. All the above evidences showed the relationship between mast cells and neuroinflammation.

BBB, the highly specialized endothelial cell membrane in the brain, represents the interface between circulating immune cells and neural cells. The BBB operates within neurovascular unit (NVU), which includes the neurons, pericytes, and clusters of glial cells. Thus, the breakdown of BBB participates in the occurrence and development of neuroinflammation during neurodegenerative diseases [2, 20]. Activated mast cells can release mediators which potentially increase the permeability of BBB, through degradation of tight junction proteins, such as MMP-9 [21]. Our previous studies showed that tryptase, the most abundant mast cell secretory product, can trigger brain microvascular endothelial cell activation via PAR-2 [8]. In the present study, we observed that cromolyn treatment significantly improves the BBB at the level of the hippocampus compared with vehicle treatment in WT mice. However, the improvement was not obvious in cromolyn-treated Wsh mice versus vehicle treatment. In addition, the breakdown of BBB was significantly inhibited in Wsh mice at 1 day but not 3 days after surgery compared with WT mice. These results indicate an early adverse effect of mast cells on BBB after surgery.

Microglia, the principal resident immune cells of the brain, are involved in brain homeostasis and neurodegenerative diseases [1]. Microglial activation has been proved to trigger neuroinflammation and neuronal damage [22]. Our previous studies showed that the activation of microglia participates in surgery-induced and LPS-induced neuroinflammation, which can be improved by intralateral ventricular microinjection of cromolyn [3, 6]. With the similar results shown in this study, microglial activation and inflammatory cytokine secretion in the hippocampus of WT mice at 1 day after the tibial surgery could be inhibited by mast cell stablization with pharmacological method. But the cromolyn sodium had no further improvement role in Wsh mice after surgery. With the similar baseline level of microglial activation and neuroinflammation, the activation of microglia and proinflammatory cytokine secretion after surgery was partially inhibited in the Wsh group. These results were then confirmed via testing the phenotype of microglia in the hippocampus after surgery. And we found that surgery obviously induced the increasing expression of M1 phenotype markers (CD86 and CD32) in the hippocampus, but the levels were significantly decreased in Wsh mice. The above results further indicate the effect of mast cells on promoting BBB breakdown and microglial activation after surgery in mice.

## 6. Conclusion

In brief, the present study clarifies that peripheral surgery evokes mast cell degranulation, following with BBB breakdown and microglial activation, leading to neuroinflammation, which can be ameliorated by mast cell stabilization and deficiency. The important role of mast cells in neuroinflammation could provide a unique and new target for therapy of neuroinflammation-related diseases.

## Data Availability

The data used to support the findings of this study are available from the corresponding author upon request.
